# Humidity-Activated Ammonia Sensor Based on Carboxylic Functionalized Cross-Linked Hydrogel

**DOI:** 10.3390/s24248154

**Published:** 2024-12-20

**Authors:** Yaping Song, Yihan Xia, Wei Zhang, Yunlong Yu, Yanyu Cui, Lichao Liu, Tong Zhang, Sen Liu, Hongran Zhao, Teng Fei

**Affiliations:** 1 State Key Laboratory of Integrated Optoelectronics, College of Electronic Science and Engineering, Jilin University, Changchun 130012, China; songyp22@mails.jlu.edu.cn (Y.S.); xiayh24@mails.jlu.edu.cn (Y.X.); weizhang24@mails.jlu.edu.cn (W.Z.); yuyl23@mails.jlu.edu.cn (Y.Y.); yycui22@mails.jlu.edu.cn (Y.C.); zhangtong@jlu.edu.cn (T.Z.); liusen@jlu.edu.cn (S.L.); 2 College of Naval Architecture and Ocean Engineering, Naval University of Engineering, P.O. Box No. 076, Wuhan 430033, China; liulichao552@163.com

**Keywords:** humidity-activated ammonia sensor, urocanic acid, fast recovery

## Abstract

Owing to its extensive use and intrinsic toxicity, NH_3_ detection is very crucial. Moisture can cause significant interference in the performance of sensors, and detecting NH_3_ in high humidity is still a challenge. In this work, a humidity-activated NH_3_ sensor was prepared by urocanic acid (URA) modifying poly (ethylene glycol) diacrylate (PEGDA) via a thiol-ene click cross-linking reaction. The optimized sensor achieved a response of 70% to 50 ppm NH_3_ at 80% RH, with a response time of 105.6 s and a recovery time of 346.8 s. The sensor was improved for response and recovery speed. In addition, the prepared sensor showed excellent selectivity to NH_3_ in high-humidity environments, making it suitable for use in some areas with high humidity all the year round or in high-humidity areas such as the detection of respiratory gas. A detailed investigation of the humidity-activated NH_3_-sensing mechanism was conducted using complex impedance plot (CIP) measurements.

## 1. Introduction

The detection of ammonia (NH_3_) is of significant importance due to its widespread industrial use and the potential health hazards it presents. NH_3_ is extensively utilized in numerous industrial processes, including refrigeration, fertilizer production, and chemical synthesis, but exposure to certain concentrations can lead to severe health issues, such as respiratory problems, eye irritation, and even fatality [1,2]. Furthermore, NH_3_ serves as an important biomarker for medical conditions such as kidney disease and H. pylori infections, making its detection critical in medical diagnostics [3,4,5]. In the food industry, NH_3_ and other total volatile basic nitrogen (TVB-N) compounds, like triethylamine, are released as a result of microbial degradation, and these compounds can be used to assess food freshness [6,7]. Therefore, NH_3_ monitoring is essential across various environments, including chemical plants, medical diagnostics, and food storage facilities [8,9].

In recent decades, significant efforts have been devoted to the development of NH_3_ sensors based on different materials and sensing mechanisms. These include sensors based on metal oxides [10,11], conjugated polymers [12,13], carbon materials [14,15,16], metal–organic frameworks (MOFs) [17,18], and various 2D semiconductor materials [19,20]. Metal oxide-based sensors, such as those made from ZnO and SnO_2_, have been widely used due to their high sensitivity and stability. Conjugated polymer-based sensors, such as polyaniline (PANI) composites, also offer good sensitivity, but often suffer from poor stability under varying environmental conditions [21]. Carbon-based materials, including graphene and carbon nanotubes, have gained considerable attention for their ability to adsorb NH_3_ molecules through π-π interactions, contributing to enhanced sensing performance. MOFs and 2D materials have shown promise due to their tunable structures and large surface areas, which facilitate better NH_3_ adsorption.

Temperature, humidity, and other variables all have an impact on how well gas sensors work [22,23]. Moisture can cause significant interference in the sensor response, resulting in reduced selectivity and sensitivity [24,25,26]. It limits the applicability of these sensors in regions with persistently high humidity levels, such as tropical or coastal areas, as well as in applications that require reliable detection under high-humidity conditions, such as early-stage disease diagnosis using breath analysis [27].

Researchers have explored various solutions to reduce the influence of high humidity on gas sensors. One approach is to apply a hydrophobic coating to the surface of sensitive materials, preventing moisture in the air from interacting with the sensitive material. For instance, Zhou et al. prepared nitrogen-functionalized heterophase TiO_2_ homojunctions (N-MXene) generated from MXene Ti_3_C_2_T_x_ as the sensing layer to identify trace NH_3_. In order to provide humidity-independent features, they employed a commercial waterproof polytetrafluoroethylene (PTFE) membrane to anchor onto the sensing layer [28]. Under each relative humidity, a smaller response than in the untreated instance occurred because the insulating waterproof glue was in close contact with the partial sensor layer. Ranjith et al. prepared a metal–organic framework (MOF) on a layered MXene hybrid by tagging a ZIF-67-based MOF on layered Ti_3_C_2_T_x_ MXene and then using a surface ligand exchange process to create an extremely sensitive, humidity-tolerant chemiresistive sensor for ultra-low ppb level NH_3_ sensing. A passive shell ligand exchange reaction gives the hybridized surface a hydrophobic surface. At relative humidities of 76% and 93%, the hybridized H-MOF/MXene-based sensor sensitivity to NH_3_ was only decreased by 0.22% and 0.27% [29]. While this approach improves the stability of sensor response, the hydrophobic layer may also hinder the target gas from contacting the gas-sensitive material, reducing sensitivity. Another strategy is introducing Ce or other elements to the oxide semiconductor. Lee et al. reported that Ce_4_W_9_O_33_, both pure and Pr-doped, can exhibit humidity-independent gas sensing properties. Moisture-endurant gas sensing properties were related to surface oxygen regeneration via the hydroxyl scavenging reaction aided by abundant Ln^3+^ (Ln = Ce, Pr) in (Ce_1−x_Pr_x_)_4_W_9_O_33_ (x = 0–0.3). The working temperature of this kind of sensor is high, so it cannot work at room temperature and consumes a lot of power [30].

To address these challenges, the concept of humidity-activated room-temperature (HART) gas sensors has been introduced [31]. The sensing mechanism of humidity-activated gas sensors involves the adsorption, dissolution, and ionization of NH_3_ in sensitive materials upon exposure to moisture. Sibi et al. developed a humidity-activated NH_3_ sensor employing tungsten-doped molybdenum/reduced graphene oxide composite, and the optimized sensor demonstrated a response of 40% to 50 ppm NH_3_ at 70% RH [32]. Kumar prepared a NH_3_ sensor based on Hierarchical α-MoO_3_ according to a chemical co-precipitation method, and the sensor demonstrated a response of 93% to 600 ppm NH_3_ at 75% RH [33]. Our previous work demonstrated that incorporating organic acid into sensitive materials can significantly enhance NH_3_ adsorption and sensor sensitivity [34,35]. However, physically doping organic acids into sensitive materials often leads to instability and the redistribution of active materials under high-humidity conditions, which adversely affects the performance and reliability of the sensor.

In this work, we address this limitation by introducing carboxyl groups—acidic functional groups—into the sensitive material through a thiol-ene click cross-linking reaction. The thiol-ene reaction is usually photo-initiated, particularly for photo-polymerizations resulting in highly uniform polymer networks [36]. A humidity-activated NH_3_ sensor was prepared by introducing urocanic acid (URA) into poly (ethylene glycol) diacrylate (PEGDA). The URA functions to enhance the adsorption capacity for NH_3_, while PEGDA serves as a hydrophilic matrix to adsorb water molecules, thereby facilitating the humidity-activated sensing mechanism. Importantly, the sensor’s response and recovery speed were optimized through careful structural adjustments within the material system applied in this work, achieving faster dynamics compared to our previous designs [34,35].

The resulting sensor exhibits excellent selectivity and stability in high-humidity environments, making it suitable for applications requiring NH_3_ detection under challenging conditions. This sensor provides a viable approach for environments such as industrial ammonia leak detection, breath ammonia monitoring in medical diagnostics, and quality assurance in food storage. Furthermore, the detailed investigation of the NH_3_ sensing mechanism conducted in this study using complex impedance plots (CIPs) provides insights into the interactions between ammonia, moisture, and the functionalized sensor material, thereby contributing to a deeper understanding of humidity-activated gas sensing mechanisms.

## 2. Materials and Methods

### 2.1. Materials

Pentaerythritol tetra (3-mercaptopropionate) (PETMP) and urocanic acid (URA) were obtained from Shanghai Macklin Biochemical Co., Ltd. (Shanghai, China). Poly (ethylene glycol) diacrylate (PEGDA, MW~1000) was acquired from Shanghai Aladdin Biochemical Technology Co., Ltd. (Shanghai, China). Benzoin dimethyl ether (DMPA) was purchased from Aladdin Industrial Corporation (Shanghai, China). Methanol was acquired from Tianjin Bohuatong Chemical Products Sales Center (Tianjin, China). All chemicals were used as received without further purification. The choice of materials, particularly PEGDA and URA, was motivated by their ability to facilitate cross-linking and enhance sensor performance through improved water molecule adsorption and proton conduction.

### 2.2. Fabrication of NH_3_ Sensors

The sensitive material for the sensors was prepared using thiol-ene click chemistry, a reaction that offers high efficiency, specificity, and ease of processing, making it suitable for fabricating functional hydrogels. The polymerized monomers included PETMP, URA and PEGDA, and their molar ratios in the sensitive layers were varied as follows: 1:0.5:1.75 (S1), 1:1:1.5 (S2), and 1:1.5:1.25 (S3). The variations in the molar ratios were designed to optimize the functional properties of the sensor, including response time, recovery time, and sensitivity.

**Figure 1:**
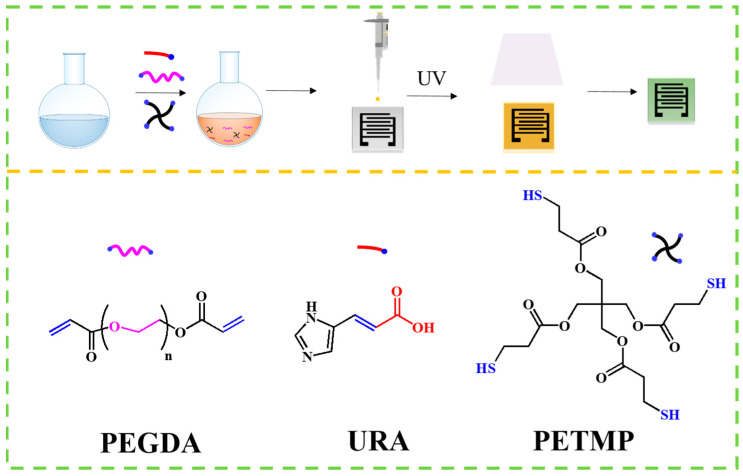
Schematic diagram for the preparation of NH_3_ sensors and the structure of monomers.

A solution was prepared by dissolving the three monomers along with the photoinitiator (DMPA) in methanol, with the reagent dosages listed in Table 1. The concentration of DMPA is 2% of the number for sulfhydryl groups. After that, 3 μL of the solution was drop-cast onto graphite interdigital electrodes on an alumina substrate (7 mm × 5 mm), which contained three electrode pairs. The electrodes were coated with the precursor solution and subjected to UV irradiation at 365 nm for 30 min with an energy intensity of 0.12 J/cm^2^ to initiate cross-linking, and form the sensitive layer (the distance from the UV source is 16 cm). After natural drying at room temperature for 8 h, the sensors are prepared. The thickness of sensitive layer is ~9 µm. The prepared process is shown in Figure 1. The use of UV irradiation for cross-linking ensures the formation of a robust network structure, which is essential for maintaining sensor stability under varying humidity conditions.

### 2.3. Measurements

The gas atmospheres were controlled by a custom-built dynamic gas distribution system. The target gas was prepared by mixing the sample gas with humidified and dry nitrogen. Humidified nitrogen was generated by passing nitrogen gas through deionized water, ensuring a consistent humidity level in the testing environment. The target gas concentration and humidity were adjusted by regulating the flow rates of sample gas, humidified nitrogen, and dry nitrogen using mass flow controllers. The target gasses included NH_3_, CH_3_OH, CH_3_COCH_3_, and C_2_H_4_.

The electrical properties of sensors were measured using a Keysight E4980AL impedance analyzer. The impedance modulus of sensors was measured at AC 1 V, 1 kHz. Complex impedance plots (CIPs) were recorded over a frequency range of 20 Hz to 1 MHz. The measurement temperature was controlled at 25 °C using an air conditioner to ensure consistent environmental conditions, as temperature fluctuations could influence the sensor response.

The response (*R*) of the sensor was defined as

(1) $$R=\frac{Z_{a}-Zg}{Zg}\times 100\%$$

where *Z_g_* and *Z_a_* represent the impedance modulus of sensors in nitrogen with and without target gas, respectively, under the same humidity conditions. The response and recovery time were denoted as the duration required by the sensor to attain 90% of the impedance modulus change for both adsorption and desorption process, respectively. The recovery time, in particular, was optimized by adjusting the cross-linking density and functional group distribution within the hydrogel matrix.

## 3. Results and Discussion

**Figure 2:**
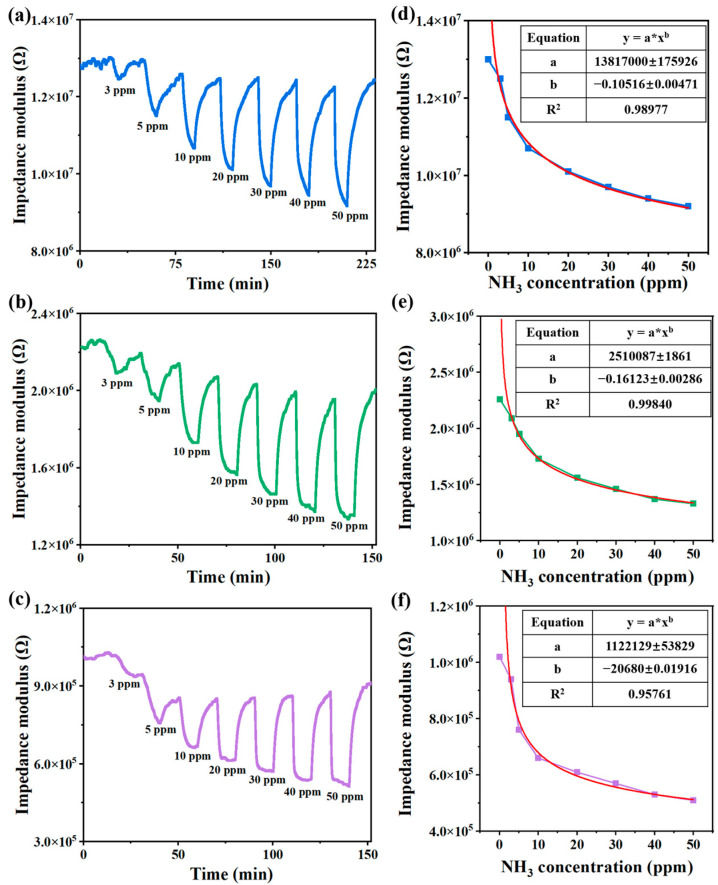
Response and recovery curves of the (**a**) S1 sensor, (**b**) S2 sensor and (**c**) S3 sensor to NH_3_ with concentrations of 3 ppm, 5 ppm, 10 ppm, 20 ppm, 30 ppm, 40 ppm, and 50 ppm at 80% RH; the relationship between the impedance modulus and ammonia concentration of the (**d**) S1 sensor, (**e**) S2 sensor, and (**f**) S3 sensor (the red line is the fitted curve of the relationship between impedance modulus and ammonia concentration).

The response and recovery characteristics of sensors (S1, S2, and S3) to different concentrations of NH_3_ (3–50 ppm) at 80% RH are shown in Figure 2a–c. The impedance modulus of sensors decreases with increasing NH_3_ concentration. A fitted equation describing the relationship between impedance modulus and NH_3_ concentration is shown in Figure 2d–f. The relationship between impedance modulus and NH_3_ concentration is consistent with the equation: y = a × x^b^. The response increased with increasing NH_3_ concentration for all sensor compositions, indicating that the adsorption of NH_3_ molecules was dependent on concentration. Table 2 summarizes the response of sensors to different NH_3_ concentrations. As the urocanic acid content increases, the sensor’s sensitivity gradually improves, but conversely, Baseline drift becomes larger. In addition, the equation for the relationship between the impedance modulus and ammonia concentration of S2 sensor fits better. Considering the response and recovery characteristics, the S2 sensor was selected for the subsequent study.

The superior performance of the S2 sensor can be attributed to its well-balanced composition. The inclusion of URA contributes to effective NH_3_ adsorption through hydrogen bonding and acid–base interactions, while PEGDA provides a hydrophilic network that supports water adsorption and proton conduction. This combination allows for an effective humidity-activated sensing mechanism, enhancing the sensitivity of the sensor, particularly under high humidity conditions.

**Figure 3:**
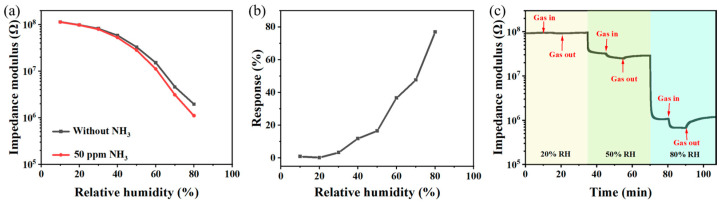
(**a**) The impedance modulus of the S2 sensor at with and without 50 ppm NH_3_ in 10–80% RH. (**b**) The response of the S2 sensor to 50 ppm NH_3_ in 10–80% RH. (**c**) The dynamic response curve of the S2 sensor to 50 ppm NH_3_ at 20% RH, 50% RH, and 80% RH.

In order to investigate the influence of humidity on the performance of the S2 sensor, the response of the S2 sensor to 50 ppm NH_3_ at 10–80% RH was evaluated. Figure 3a shows the impedance modulus of the S2 sensor in the atmosphere with and without 50 ppm NH_3_ in 10–80% RH, and Figure 3b indicates the response of the S2 sensor to 50 ppm NH_3_ in the same humidity range. As can be seen, the impedance modulus of the S2 sensor remained almost unchanged when relative humidity was below 30%, indicating negligible response. However, above 30% RH, the impedance modulus of the S2 sensor decreased after introducing 50 ppm NH_3_, resulting in an increased sensor response. Figure 3c presents the dynamic response curve of the S2 sensor to 50 ppm NH_3_ at 20% RH, 50% RH, and 80% RH. It is noteworthy that the sensitive layer reacted with NH_3_ at 50% RH and 80% RH, but not at all to 50 ppm NH_3_ at 20% RH.

**Figure 4:**
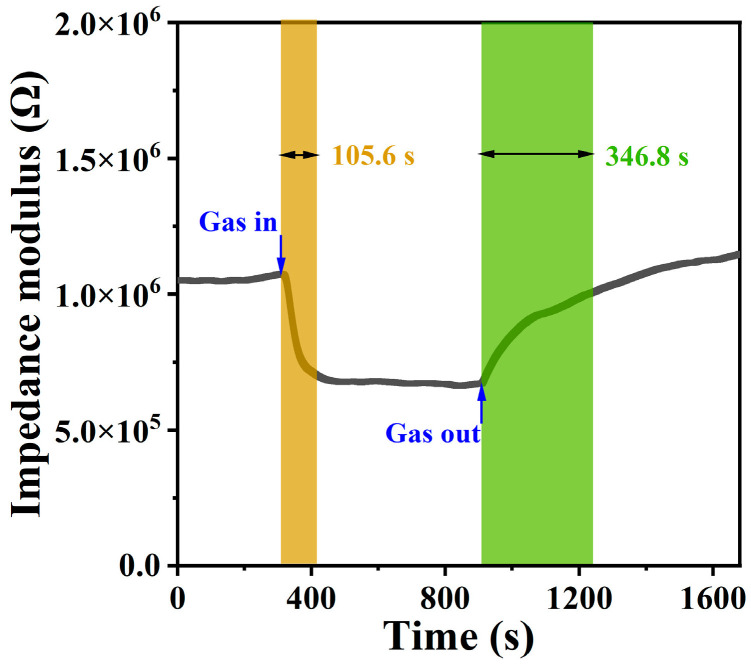
Response and recovery curve of S2 sensor upon exposure to 50 ppm NH_3_ at 80% RH.

When assessing the performance of a gas sensor, the response/recovery time is an important parameter. Figure 4 depicts the response and recovery curve of the S2 sensor upon exposure to 50 ppm NH_3_ at 80% RH. The response time was measured to be approximately 105.6 s, while the recovery time was about 346.8 s. The properties of some reported NH_3_ sensors are listed in Table 3. Compared to published NH_3_ sensors, the S2 sensor exhibits a relatively short recovery time.

**Figure 5:**
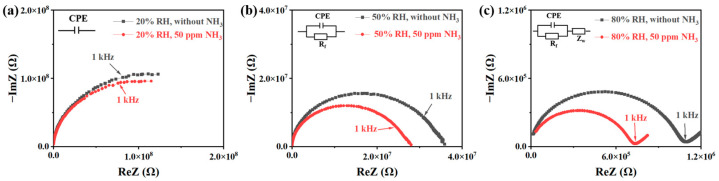
Complex impedance plots of S2 sensor without NH_3_ (gray) or 50 ppm NH_3_ (red) at (**a**) 20% RH, (**b**) 50% RH, and (**c**) 80% RH.

As shown in Figure 5, in order to explore the sensing mechanism of the sensor, the complex impedance plots (CIPs) of the S2 sensor were measured before and after introducing NH_3_ at different humidity levels: 20% RH, 50% RH, and 80% RH. The real part (ReZ) and imaginary part (ImZ) of the complex impedance represent the resistive and reactive characteristics of the material, respectively. As illustrated in Figure 5a, the CIP shows an incomplete semicircle arc at 20% RH, both in the presence and absence of NH_3_. This pattern suggests that the equivalent circuit is composed of a parallel resistor (R_p_) and a capacitor (C_p_), with the primary conductive channel functioning as a capacitance. At 20% RH, the conductive path is discontinuous within the sensitive material, and the conduction mechanism is primarily limited to proton hopping.

As demonstrated in Figure 5b, the CIP at 50% RH exhibits a distinct semicircle in both the presence and absence of NH_3_, which indicates that the equivalent circuit still consists of a parallel R_p_ and C_p_ configuration. However, at this stage, the primary conductive channel shifts from capacitance to resistance, indicating that a complete conductive path has formed within the sensitive material. The sensitive material adsorbs enough water molecules to create a continuous water film at 50% RH, allowing protons to transfer to adjacent water molecules via the Grotthuss mechanism. Nonetheless, the amount of water present in the sensitive material is still insufficient to support large-scale electrolyte dissociation and ionic conduction at 50% RH.

As reflected in Figure 5c, the CIP at 80% RH displays a semicircle with an additional straight line at low frequencies. This feature demonstrates the presence of Warburg impedance (Z_w_), indicating ion diffusion from the sensitive material and the electrode. At 80% RH, the sensitive material adsorbs enough water molecules to support electrolyte dissociation and ionic conduction. Under these conditions, the main conduction mode of the sensor is ionic migration, facilitated by the high availability of water molecules that support continuous proton and ion conduction pathways.

**Figure 6:**
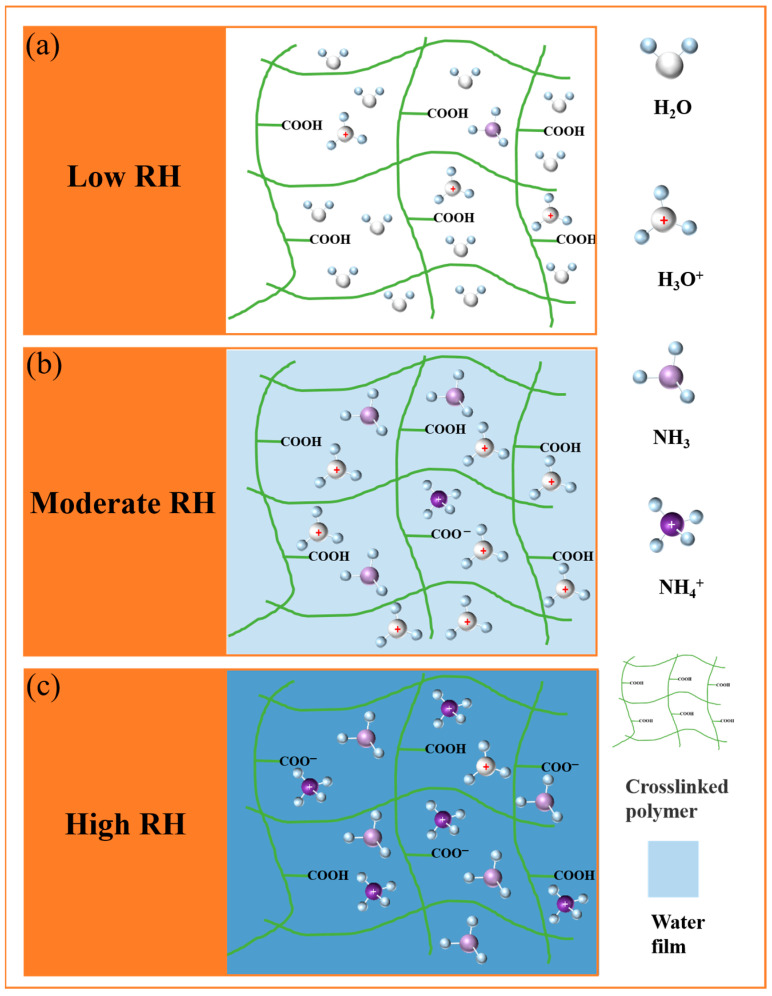
Proposed conduction mechanisms in the S2 sensor in the presence of humidity and NH_3_: (**a**) at low relative humidity (RH), (**b**) at moderate RH, and (**c**) at high RH.

As depicted in Figure 6, the CIPs and primary conductive channel of the S2 sensor can be divided into three categories, each corresponding to a different sensing process operating under varying humidity levels. At 20% RH, the primary conductive channel functions as a capacitance, which remains consistent even with the introduction of NH_3_ (Figure 5a). Under low relative humidity (RH) conditions, as shown in Figure 6a, the number of water molecules adsorbed inside the sensitive membrane is limited, resulting in a discontinuous proton conduction path. As humidity increases to 50% RH, the primary conductive channel shifts from capacitance to resistance, regardless of NH_3_ exposure (Figure 5b). At this level of humidity, a complete conductive path forms inside the sensitive material, allowing for proton transmission along a continuous water film.

However, under moderate RH conditions, the sensitive material adsorbs enough water molecules to form a water film that enables continuous proton conduction. At this stage, the material does not yet support large-scale electrolyte dissociation and ion migration, and the conduction mechanism is dominated by proton hopping (Figure 6b). At 80% RH, the appearance of Warburg impedance at low frequencies indicates that ion migration occurs between the sensitive material and the electrode (Figure 5c). Under these high-humidity conditions, ion migration becomes the dominant mode of conduction. The sensitive material adsorbs enough water molecules to support the dissociation of electrolytes and facilitates ion migration, leading to a marked increase in sensor performance (Figure 6c).

The chemical reactions involved in the sensing mechanism under high RH conditions include the auto-ionization of water and interactions between the carboxyl groups and NH_3_, which are described below:

H_2_O (ads) + H_2_O (ads) ⇌ H_3_O^+^ + OH^−^ (2)

RCOOH + H_2_O (ads) ⇌ RCOO^−^ + H_3_O^+^ (3)

NH_3_ + H_2_O (ads) ⇌ NH_4_^+^ + OH^−^ (4)

RCOOH + NH_3_ (ads) → NH_4_^+^ + RCOO^−^ (5)

**Figure 7:**
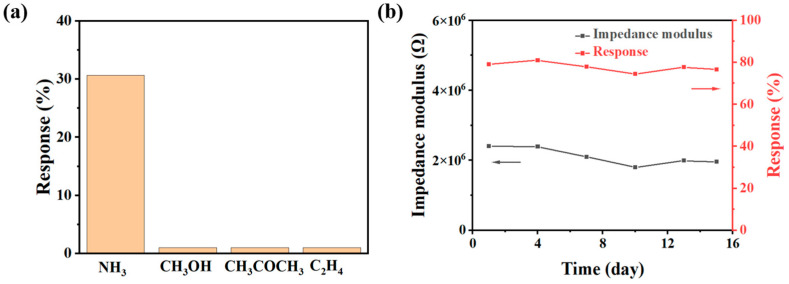
(**a**) The response of the S2 sensor to 10 ppm NH_3_, CH_3_OH, CH_3_COCH_3_, and C_2_H_4_ at 25 °C at 80% RH. (**b**) The impedance modulus (black) of the S2 sensor at 80% RH and the response (red) of the S2 sensor to 50 ppm NH_3_ at 80% RH over 15 days.

Selectivity is one of the most important functions for gas sensors, as it defines the sensor’s ability to detect the target gas in the presence of other gasses. The response of the S2 sensor to a range of gasses, including NH_3_, CH_3_OH, CH_3_COCH_3_, and C_2_H_4_, at 80% RH, is displayed in Figure 7a. The response of the S2 sensor to NH_3_ is significantly greater than that of the other gasses, indicating that the S2 sensor possesses excellent selectivity. The selective response can be attributed to the specific interactions between NH_3_ molecules and the functional groups (e.g., carboxyl groups) in the sensing material, which are not present for the other gasses tested. These interactions enhance the sensor’s sensitivity specifically to NH_3_ while minimizing the response to other gasses, even under high-humidity conditions.

Long-term stability is another important characteristic of a sensor, as it reflects the ability of the sensor to function consistently over an extended period. Figure 7b shows the impedance modulus of the S2 sensor at 80% RH and its response to 50 ppm NH_3_ over a period of 15 days. As shown, the impedance modulus of the S2 sensor at 80% RH, as well as its response to 50 ppm NH_3_ (an error of approximately 2.8% over 15 days), remains relatively unchanged over the 15-day period, demonstrating that the sensor maintains good long-term stability. This stability is crucial for practical applications, as sensors often need to operate under varying environmental conditions for prolonged periods.

The stability of the S2 sensor can be attributed to the robustness of the cross-linked hydrogel structure, which prevents significant changes in the material properties over time. The in situ photoinitiated polymerization process used to create the cross-linked PETMP-PEGDA-URA structure results in a stable network that is less prone to degradation or reorganization when exposed to moisture, thus ensuring long-term performance consistency.

## 4. Conclusions

Using an in situ photoinitiated polymerization approach, a novel PETMP-PEGDA-URA humidity-activated NH_3_ sensor was successfully prepared. The cross-linked network within the sensitive material provides the sensor with remarkable stability and enhanced performance in high-humidity environments. The optimized sensor achieved a response of 70% to 50 ppm NH_3_ at 80% RH, with a response time of 105.6 s and a recovery time of 346.8 s. The sensor demonstrated a strong selectivity for NH_3_ and a fast recovery in high humidity conditions, making it suitable for use in environments where humidity is a significant factor. A detailed investigation of the NH_3_ sensing mechanism using complex impedance plots revealed that the sensor operates through different conduction mechanisms depending on the humidity level. At low RH, the primary conduction mechanism is proton hopping, while at moderate RH, proton conduction occurs through a continuous water film. At high RH, ion migration dominates due to sufficient electrolyte dissociation. The analysis of chemical reactions showed that NH_3_ interacts with water and carboxyl groups within the sensor, leading to increased ion production and enhanced conductivity.

## Figures and Tables

**Table 1:** Dosage of reagents in solution of corresponding sensor.

Sensor	PETMP (mg)	URA (mg)	PEGDA (mg)	DMPA (mg)	Methanol (mL)
S1	50.0	7.4	179.0	2.1	4.0
S2	50.0	14.9	153.5	2.1	4.0
S3	50.0	22.3	127.9	2.1	4.0

**Table 2:** Response of the sensor to corresponding NH_3_ concentration at 80% RH.

NH_3_ Concentration	Response (S1 Sensor)	Response (S2 Sensor)	Response (S3 Sensor)
3 ppm	4.8%	8.1%	7.4%
5 ppm	13.0%	16.5%	32.9%
10 ppm	22.0%	30.6%	53.0%
20 ppm	28.7%	43.9%	65.6%
30 ppm	34.3%	54.8%	77.1%
40 ppm	37.4%	63.7%	87.7%
50 ppm	42.9%	70.0%	94.2%

**Table 3:** The properties of some reported NH_3_ sensors and the S2 sensor in this work.

Sensing Material	Response (%)	Response/Recovery Time (s)	Operating Condition	Ref.
PH-Co_3_O_4_	280 (100 ppm)	450/650	RT/50% RH	[37]
RGO	80 (10 ppm)	31/500	RT/50% RH	[38]
SnO_2_	286 (20 ppm)	~575/~563	RT/70% RH	[39]
rGO/Co_3_O_4_	2 (100 ppm)	351/1199	RT/51% RH	[40]
Graphene aerogel	80 (90 ppm)	60/500	RT/45% RH	[41]
PETMP-PEGDA-URA	70 (50 ppm)	106/347	RT/80% RH	This work

## Data Availability

The data are contained within this article.
